# Ultrasonic Sensors Enabling Early Detection of Emergency Trends and Analysis of Structure Inclination and Stability by Means of Highly Accurate Level Measurements

**DOI:** 10.3390/s21051789

**Published:** 2021-03-04

**Authors:** Leszek Ornoch, Paweł Popielski, Andrzej Olszewski, Adam Kasprzak

**Affiliations:** 1 Ultrasystem, Nowolipki 36/18, 01-019 Warsaw, Poland; ornoch_xl@wp.pl (L.O.); andrzej.olszewski@gmail.com (A.O.); 2 Faculty of Building Services, Hydro and Environmental Engineering, Warsaw University of Technology, Nowowiejska 20, 00-653 Warsaw, Poland; adam.kasprzak@pw.edu.pl

**Keywords:** precise sensors, long-term stability, SHM

## Abstract

Building inclinations can be measured through the use of ultrasonic hydrostatic levelers. These are used to measure long-term relative displacements of vertical parts of structures and utilize the principle of communicating vessels (similar to the classic water scales). The presented ultrasonic displacement measurement technique was developed by Ultrasystem in the 1990s and was applied to several objects in Poland. Long-term measurements enabled the development of a model of object behavior under the influence of various factors. Among these are the annual cycle of temperature changes, fluctuating water levels, turbine chamber emptying, etc. Such a model can facilitate the prediction of failure based on the appearance of changes deviating from typical behavior (e.g., a much stronger dependence of the inclination as a function of the water level). The results obtained with the help of ultrasonic sensors enable the observation of subtle deformations of the object, which is valuable when developing and calibrating new models of the object (e.g., by means of the finite element method).

## 1. Introduction

A feeling that inevitably accompanies design, construction and the use of each and every structure, including hydraulic structures, is that of limited and unavoidable uncertainty—the uncertainty resulting from the limited accuracy of ground exploration or related with the intensity of the effects and impacts of natural or other random factors [1,2]. Hydraulic structures, the purpose of which is permanent or seasonal water damming, are exposed to ground degradation by filtration and the aging of construction materials [3]. Their design must also take into account exposure to extreme natural conditions, such as hydrological or seismic phenomena.

However, this uncertainty is offset by the so-called safety margin—the result of load factors and construction material parameters assumed for the design—which is guaranteed at all the stages of construction and use [4]. Thanks to the safety margin factor, it is possible to apply risk management procedures, both during the structure construction and operation. The purpose of the risk management procedure is to make the most efficient use of the safety margin, depending on the current configuration and intensity of the effects to which the object is exposed, and if the margin is near to being breached to take appropriate protective measures in advance. Precise risk assessments require reliable and sound assumptions that can be made only if the monitoring system is reliable and has been designed appropriately. Such a system should guarantee the control of processes that have a material impact on the safety of the structure and its direct environment, while observing a required level of accuracy. Its dependability should guarantee reasonable (structural) redundancy to enable the mutual verification of the observation results during the structure’s operation [5].

The monitoring system should be capable of warning, as well as providing data, for developing better models of the object behavior. The warning function is based mostly on standardized procedures that aim at delivering information about the current load of the structure and the ground and about the response to external effects, according to the quantitative criteria ensured by the designer. Those criteria usually take the form of threshold values (e.g., information threshold, warning threshold and emergency threshold), which are linked to the characteristic and design values of designed loads and/or conditions of structure serviceability. Achieving or exceeding particular thresholds is usually related to the routine launch of certain standard and necessary preventive actions, notification and warning procedures, etc.

By means of the data providing a function, one can ascertain whether ongoing monitoring processes correspond with the design assumptions, and in the case of detecting major deviations, update forecasts. In addition, one can verify the current and future safety levels, indicate possible hazards and suggest necessary preventive measures [6,7]. In order for the function to be fulfilled properly, the designer, contractor and servicing staff have to strictly collaborate on a long-standing basis. The scope of that collaboration includes the verification and assessment of multiyear monitoring results, the assessment of possible hazards and—if necessary—the formulation of detailed recommendations concerning the necessary changes in the facility operating plan, the periodic limitation of service, modifications of the monitoring system, etc. [8]. The key tasks of the expert team include conducting regular visual inspections, interpreting the observation results on an as-is basis and explaining the causes of material deviations from the projected values, as well as updating the threshold values for the purposes of the warning function. The importance of these tasks for ensuring the safety, continuous serviceability and elimination of losses caused by possible failures grows proportionally to the level of uncertainty in terms of the ground examination and gradual changes of its parameters, technical conditions and sensitivity of construction, in addition to the possible values and mutual configurations of the short-term and long-term variable loads [7].

The ultrasonic technique of displacement measurement, presented herein, was developed by Ultrasystem (www.ultrasystem.eu, accessed on 1 March 2021) in the 1990s. This method utilizes a principle of operation that is similar to that applied in the scientific echosounder [9]. Modifications involving the application of specially designed ultrasonic transducers, as well as the adoption of new solutions in terms of electronic system configuration, has resulted in the achievement of unique microscale accuracy and extremely high-level long-term stability.

Advantages of the method include the broad-scale measurement of the liquid levels (from single millimeters to many meters), very high resolution and accuracy, linear characteristics within the entire measurement range, and the absence of mechanical moving elements in the sensor construction (hereby negating the zero drift and the hysteresis effects of other types of sensors) [10].

Observations and tests conducted between 1990—2019 at various facilities and in laboratory conditions on more than 100 sensors have provided evidence that the unique long-term stability of UMP3 (ultrasonic inclinometer) is better than one arcsec/year and the resolution better than 0.01 arcsec (arcsec—5 µm/m) for the inclination measurements (with vessel distances of 1–3 m). Such sensors have been installed in a number of hydraulic facilities in Poland [10]. Within a dozen recent years, the inclination observed at these facilities has never been greater than 0.1° (typically, 0.02°) and, in the case of annual and circadian cycles, 0.01°. The data obtained with the use of the sensors, as well as the principle of result compilation, indicate that the sensors feature very high accuracy and stability. That is why the principle of the sensors operation and the measurement results processing have been described in detail in this paper.

## 2. Materials and Methods

The UMP3 ultrasonic hydrostatic leveler can measure various construction shifts and tilts. The device is based on the classical water scale that was established on the principle of communicating vessels. It is used to measure the long-term relative displacements of the vertical parts of structures.

In each vessel of the UMP3 device, the height of the fluid column is determined by measuring the ultrasonic pulse travel time. The speed of sound within the fluid depends on the temperature. In order for the measurement result to not be influenced by temperature fluctuations, two channels are used within the measurement system. One channel measures the distance of the transducer from the fluid surface, and the second, called the “reference channel”, measures the known, fixed distance. In addition, the reference channel enables a very precise measurement of the temperature inside the device without any additional thermometer being incorporated within the system.

Due to the unique and proprietary concept of an ultrasonic transducer, as well as specially designed electronics, it is possible to directly measure the level of the fluid in every vessel with exceptionally high sensitivity (0.0001 mm). This outcome is unprecedented in other devices of this type. While taking into account the distance between the two measuring vessels, the changes of the tilt of the monitored surface can be measured with a sensitivity as high as 0.02 arcsec [10].

The absence of flexible elements, as well as meticulously designed manufacturing technology, make it possible to achieve state-of-the-art long-term stability. Our sensors have proven their quality serving various structures for over 20 years. Surveys over many years have confirmed that the parameters of the measurements performed by our devices reach 0.005 mm per annum in the case of the UMP3 leveler and 0.005 mm/m per annum for the inclinometer.

This article compares the declared stability parameters of the best mechanical inclinometers with the stability parameters of our ultrasonic UMP3 sensors, as determined from long-term measurements performed on an actual water dam. Two types of mechanical inclinometers achieving the highest long-term measuring stability have been selected for this analysis [11].

The most common solutions on the market enable the automatic measurements of vertical displacements along monitored constructions [12]. They apply the communicating vessels concept, which enables measurements of the fluid levels or pressure. Based on that, it is possible to determine the position of each of the vessels in relation to the reference chamber attached to the stable surface. Such a design allows for precision only as high as 0.1 mm, placing it far behind the ultrasonic UMP3 solutions presented in this article, as far as the sensitivity of the measurements is concerned. The other manufacturer, unlike Ultrasystem, does not provide long-term stability parameters.

Obtaining the long-term results of extremely high resolution and precision enables the extraction of every subtle change of the shape or the rotation of the construction, based on periodic changes, and allows for the correlation of them with the influence of external factors (e.g., meteorological circumstances) and maintenance loads. An assurance of the stability and precision of the measurements enables the forecast of future deformation processes of the building or the object. Furthermore, by utilizing the appropriate software tools, it is then possible to foresee displacement trends, as well as the occurrence of potentially dangerous states, long before reaching alarm levels.

## 3. Ultrasonic Sensor Operation

**Figure 1:**
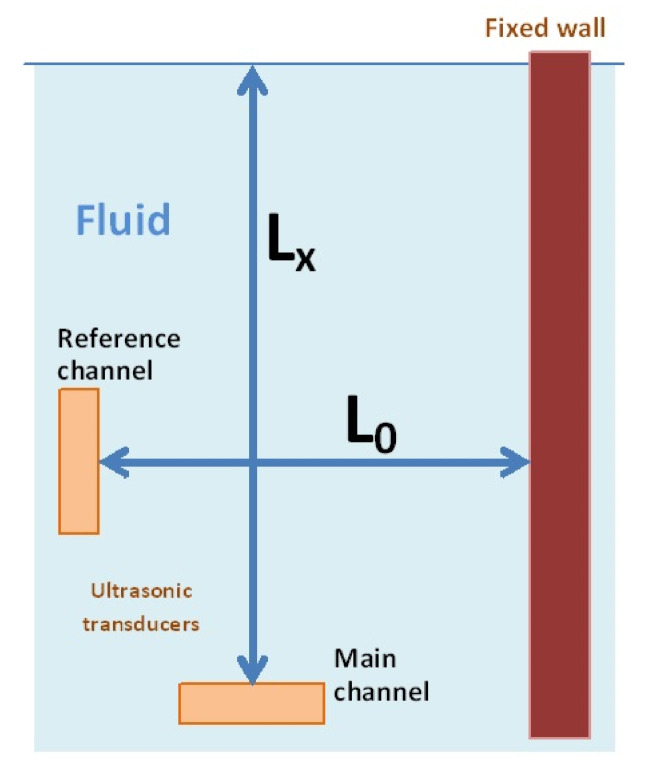
Diagram of the level sensor used in the UMP3 leveler device.

In the device (Figure 1), the distance Lx between ultrasonic transducer and free surface is determined by measuring the ultrasonic pulse travel time. A vibrating transducer passes this ultrasonic pulse to the fluid chamber. Propagating with the speed of sound V, the pulse reaches the free surface, from which it reflects and returns to the transducer after the following time [13]:

Tx = 2∙Lx/V

The speed of sound within the fluid depends on the temperature; hence, so that the measurement result of distance L is not conditioned by the effect thereof, two channels are used within the measurement system [14]. One channel measures the distance Lx of the transducer from the free surface, and the second—the reference channel—measures the known distance Lo between the second transducer and the fixed wall.

Tx = 2∙Lx/V

To = 2∙Lo/V

Thus, distance Lx equals

Lx = Lo∙Tx/To

The above formula for determination of distance Lx does not openly depend on the speed of sound. This results from an assumption that it is equal in both measurement channels. This condition is fulfilled with great accuracy provided the device is constructed appropriately—namely, so that it minimizes the occurrence of temperature gradients. This is achieved by maintaining symmetry for both channels and by surrounding the measurement area with a thick layer of metal (which is a good conductor of heat). That is why the ultrasonic sensor is placed inside a measurement vessel featuring an external diameter of 105 mm and a wall thickness of 10 mm and is suspended from the upper cover on elements featuring low thermal conductivity. Such a construction ensures the minimization of the temperature difference between the measurement channel and the reference channel and guarantees the assumed accuracy.

## 4. Temperature Measurement in Ultrasonic Sensors

The use of two measurement channels makes it possible to determine the temperature inside the leveler without incorporating an additional thermometer. This is very practical in many ways. Thanks to this solution, it is possible, e.g., to compensate for the effect of fluid thermal expansion (which is especially important for hydrostatic levelers) and to establish the result/temperature correlations. In the reference channel at the set distance Lo between the transducer and the fixed wall, the pulse travel time can be calculated as follows:

To = 2∙Lo/V

The speed of sound is a function of temperature. For example, for the fluid used in the device, within the range of temperatures concerned, this is a linear function of the temperature, and

V = Vo∙(1 + α∙temp)

where:

Vo—speed of sound at 0 °C,

α—temperature-dependent speed of sound change ratio and

temp—temperature in °C.

Having determined the To value, one can calculate the temperature while applying the following formula

temp = (1/α)∙(To(0 °C)/To − 1)

where To (0 °C) means travel time at 0 °C.

The α and To (0 °C) parameters, which are fixed values characterizing the sensor, are ascertained during the device calibration procedure.

The following are some parameters of the fluid level measuring sensor UMP3:

- measurement range—40 mm,
- resolution—0.1 μm (0.0001 mm),
- long-term stability (1 year)—5.0 μm (0.005 mm) and
- operating temperature—−20 °C + 50 °C.

## 5. Ultrasonic Leveler Operating Principle

**Figure 2:**
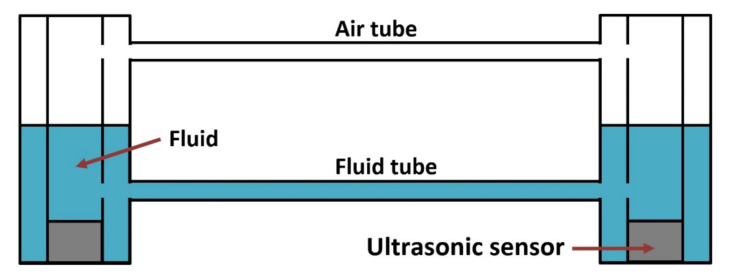
Hydrostatic leveler UMP3.

The ultrasonic leveler, the diagram of which is presented in Figure 2, is based on the principle of communicating vessels and is used for measuring the deformation of structure fragments. It is composed of measuring vessels connected with flexible plastic tubes. The lower tube is filled with fluid, and its role is to balance the fluid level in the vessels; the upper tube ensures equal air pressure in both vessels.

Each measurement vessel is equipped with fluid level measuring sensors with precise temperature measurements. This enables thermal expansion compensation in the case of temperature differences between the sensors. By the fluid level measurements, vertical displacement occurring between the vessel mounting points can be determined. If both vessels are mounted to a uniform block (e.g., concrete or stone structures) the leveler plays the role of an inclinometer, the base of which is determined by the distance between the vessels. In the case of classical inclinometers featuring compact construction, the stability of the mounting screws by means of which the sensor is fixed to the ground is a real problem due to the narrow spacing of the mounting screws (typically, approx. 10 cm). In inclinometers composed of two measurement vessels that are located within a significant distance from each other (typically from 1 m to 5 m), this problem is rather negated.

## 6. Temperature Impact Compensation

**Figure 3:**
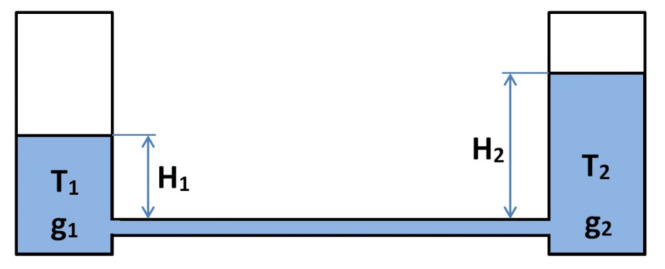
Level measuring vessels in the case of a temperature difference. H_1_ and H_2_—fluid column height above the fluid tube, g1(T1) and g2(T2)—fluid density for temperatures T1 and T2.

If a temperature difference occurs between the measurement vessels, the fluid surface in one vessel cannot balance that in the other vessel due to the thermal expansion and resulting differences in the fluid density. In order to achieve a high accuracy, this phenomenon is taken account of by using the computer software that controls the leveler. The software processes the results of the temperature measurements in each sensor. A case of measurement vessels featuring different temperatures is presented in Figure 3.

The balance between both vessels is conditional upon equal hydrostatic pressure at the fluid tube level (no flow observed between the vessels); thus:

g_1_(T_1_)∙H_1_ = g_2_(T_2_)∙H_2_

where:

g_1_(T_1_) and g_2_(T_2_)—fluid density for temperatures T_1_ and T_2_, and

H_1_ and H_2_—fluid column height above the fluid tube.

The fluid density within the temperature function is determined by the following formula:

g(T) = go∙(1 + β∙ T)

where:

go—fluid density at 0 °C, and

T—temperature in °C.

Ultrasonic sensors measure the actual fluid levels H_1_ and H_2_, which—for the purpose of calculation—must be reduced to the same temperature, e.g., to 0 °C. To this end, the intended fluid level must be divided by (1 β∙T); thus:

H_reduced_ = H_measured_/(1 β∙T)

If the fluid tube does not run horizontally and the temperature is distributed across the whole length thereof, an insignificant difference of levels in the vessels may occur due to the different fluid density. This is why, when mounting the leveler, it is important to make sure that the fluid tubes are in a horizontal position all the time.

## 7. Fluid Balance Setting Period

The hydrostatic leveler is intended for detecting vertical displacements between the measuring vessels. After such movement occurs, a certain time is necessary for a new balance to be established. For ensuring the appropriate observation of the changes, it is important that the balance-setting period is shorter than the time interval required between the subsequent measurements.

**Figure 4:**
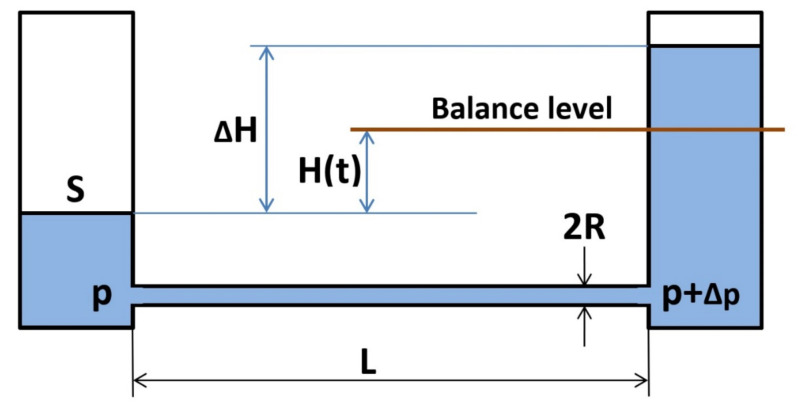
Reaching the balance of the liquid levels in the leveler vessels.

For the purposes of determining the balance-setting period, it is assumed that a certain displacement occurred that caused a ∆H difference of the fluid levels in the vessels. The result of this is that the fluid started to flow from one vessel to the other (Figure 4). The volume of the fluid flowing in a unit of time through a long circular cross-section, featuring a ∆p difference in the pressure between both ends, is determined by means of the Poiseuille equation [15]:

Q = ∆p (π R^4^)/(8 η L)

R—pipe radius,

L—length of the pipe and

η—dynamic viscosity.

The momentary difference in pressure equals:

∆p(t) = 2 ρ g H(t)

ρ—fluid density,

g—gravitational constant and

S—surface area of the fluid inside the vessel.

By solving the differential equation for the flow rate:

S dH(t)/dt = Q,

we arrive at a formula that determines the balance-setting period:

H(t) = 1/2 ∆H e − t/τ

where the time constant is τ = (4 η L S)/π ρ g R^4^) (the very strong influence of the tube diameter on the balance-setting period can be visible—a twice-larger diameter results in a 16-fold time shortening!).

Depending on the type of liquid, vessel spacing and tube diameter/cross-section, the time constant may vary from approx. one second to a dozen or so minutes.

The ultrasonic measurement method applied in the leveler resulted in the achievement of a very high resolution and stability of measurement. This method is ten times more accurate than the classical methods, and this effect determines its usefulness for the examination of structures (especially made of concrete) in the long term. Indeed, due to the application of reference measurements in each of the vessels, the angle measurement characteristics do not depend on the temperature (Figure 1). This is unique to our system.

**Figure 5:**
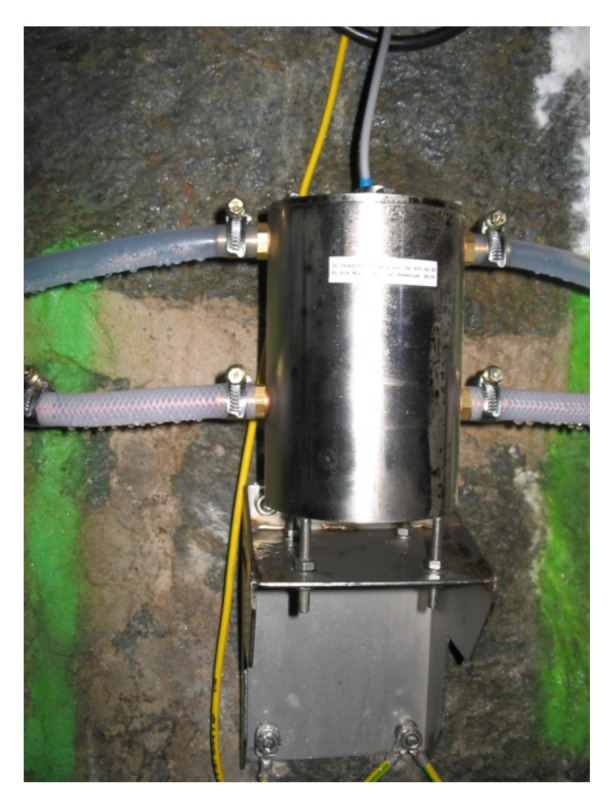
A UMP3 leveler unit with an ultrasonic sensor that was installed on one of the objects (appearance after a period of 15 years of use in harsh environmental conditions—high humidity).

Figure 5 shows one of the vessels of the hydrostatic line leveler containing UMP3 sensor operating for over 15 years on the water dam discussed in the article.

## 8. Examples of Measurement Results

**Figure 6:**
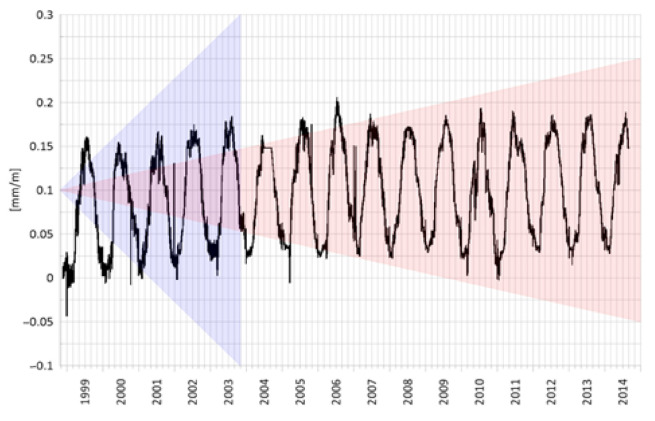
The measurement results of the inclinations of a hydroelectric power block measured by UMP3 ultrasonic sensors in the period of 15 years against the declared long-term stability of other types of inclinometers. The blue and red zones show potential zero drift zones for the respective sensors—Zerotronic 3 and Zeromatic 2/2.

In Figure 6, the black graph illustrates the results of the measurements of a hydroelectric power plant unit inclination within the time span of 15 years, as obtained by a UMP3 ultrasonic inclinometer.

Here, a clear annual cycle is visible. This is related to the thermal movement of the structure and is a very slight constant trend at the level of 0.03 mm/m throughout the whole period. In the background of this, one can observe circadian fluctuations within the range of 0.02 mm/m. For the sake of comparison, two types of levelers were selected that feature some of the best parameters of long-term stability among the currently used levelers dedicated for structure control.

The red and blue zones represent declared zero drift areas for the devices manufactured by a leading European company. The red zone shows the behavior of a reversal Zeromatic 2/2 device and the blue zone the behavior of a Zerotronic 3 device within the measurement range of ±0.5°. These levelers additionally feature a zero drift dependent on a temperature coefficient. This introduces an additional error for measuring a nonzero angle. This phenomenon is negated in ultrasonic levelers and inclinometers.

The ultrasonic sensors installed on the other structures demonstrate a similar stability as in the above graph, even within the period of 20 years. This enables determining a structure behavior model and detecting even a slight deviation from the typical behavior, thereby enabling the early detection of emergencies.

**Figure 7:**
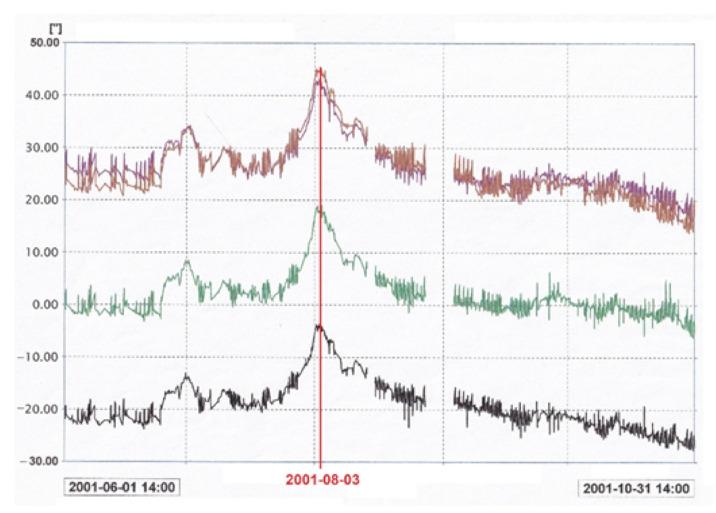
Measurements of change of inclination of 4 fragments of a hydrotechnical facility measured by a number of UMP3 units versus changes in the water level June–October 2001 (Figure 8).

**Figure 8:**
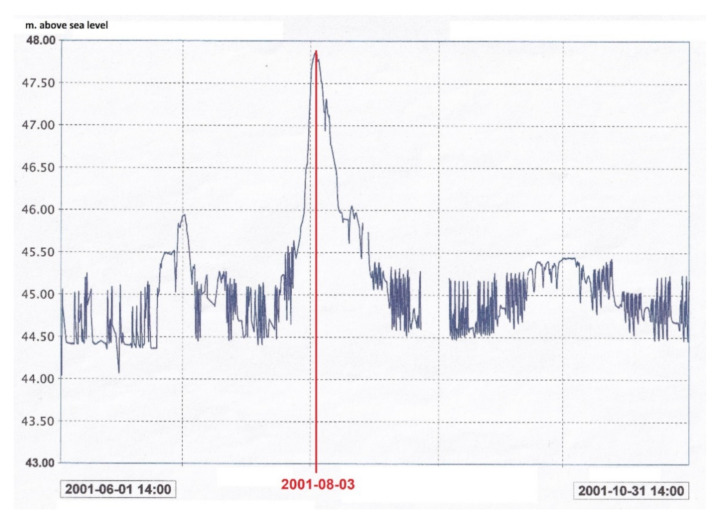
Changes in the water level in the same period of time as the observed changes in inclination (Figure 7).

The graphs in Figure 7 illustrate inclinations for four different fragments of a weir that is part of a hydraulic structure and a measured level of the tailwater in the same period of time (Figure 8). These parameters are fully correlated for all the weirs. Here, an increase of the water level by 3.5 m induced an inclination of 20 arcsec (i.e., 100 µm/m) towards the tailwater. The circadian changes of the inclination and water level are visible in the graphs and are related to the circadian rhythm of the hydroelectric plant operation.

The above-mentioned results were achieved a number of years ago, and if discrepancies occurred today (greater inclination at the same change of the water level), it could indicate, e.g., ground deformations as a result of infiltration [3,16]. In such a case, preventive measures could be taken early enough for remediation. Since there is more than one leveler, one can determine the area where the phenomenon occurs, provided that the change was indicated by one sensor alone.

## 9. The Use of Numerical Simulations to Assess the Condition of the Facility and the Location of Measurement Points

High dams creating large reservoirs require constant monitoring due to the substantial effects of a potential disaster or accident [17,18,19,20].

The factor that may support the selection of the location of displacement sensors is based on a numerical model, such as, for example, the finite element method [21,22]. At different stages of the life of the structure, we can distinguish diverse classes of models characterized by different accuracies and complexities. At the design stage, when building the model, we mainly rely on assumptions regarding the materials, soil and weather conditions, as well as the loads that will affect the object during construction and operation [23].

The design assumptions are verified during the operational stage. The calculations already take into account the shape and static scheme of the structure based on the as-built documentation and on additional geodetic inventories [24,25,26]. Models intended for design are most often based on a simplified mapping of the geometry of the object, and the constitutive models used in them must allow for the analysis of various load variants in order to design appropriate cross-sections. Most often, due to the use of a combination of loads, elastic models are employed, and the plastic performance is taken into account as factors reducing both the stiffness and strength parameters.

The model intended for the analysis of the actual behavior of the structure, the purpose of which will be to determine the displacements and stresses and to choose or verify the selected type of monitoring, should be more detailed. Instead of, for example, elastic materials used in the process of designing the model, it should take into account the plastic strains and such phenomena as creep or relaxation.

**Figure 9:**
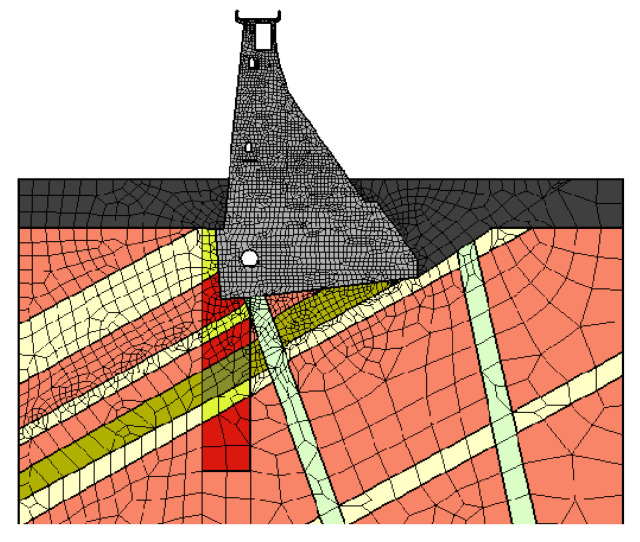
Geometry and material zones of the created numerical model.

An example of a concrete gravity dam model is shown in Figure 9, and a description of the calculations is presented below. Figure 9 shows a cross-section through the concrete dam section and soil layers present in the subsoil. Numerical calculations were performed utilizing the ZSoil program [27].

**Figure 10:**
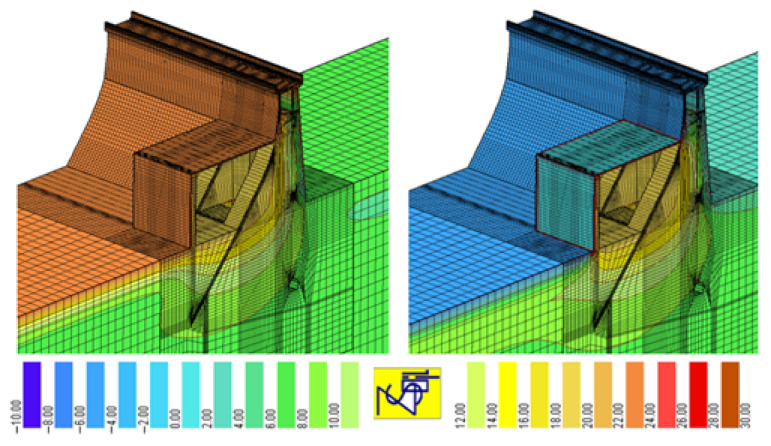
Temperature distribution inside a concrete dam in the summer and winter [30].

In concrete hydrotechnical structures, the daily and seasonal changes in the air, water and structure temperature have a significant impact on the formation of stresses and displacements [28,29]. Numerical calculations of such objects should be carried out as a one-way coupled thermomechanical analysis. The temperature field determined in the thermal analysis is then used to calculate the stresses and strains resulting from the temperature changes. Boundary conditions for the thermal analysis can be obtained from the measurements of the air temperature outside the object, in the control and measurement galleries and from the solar radiation [30]. In order to capture the effect of water cooling of an upstream face in the summer and heating in the winter, it is also necessary to determine the water temperature distribution, along with the depth in the reservoir at the dam, as it changes throughout the year. Sample results of a temperature analysis are shown in Figure 10.

After determining the temperature field, the model in which the stresses and displacements are analyzed is loaded with its own weight, pressure of the tailwater and headwater and other forces that can be caused, for example, by cranes or turbine generator sets [30].

**Figure 11:**
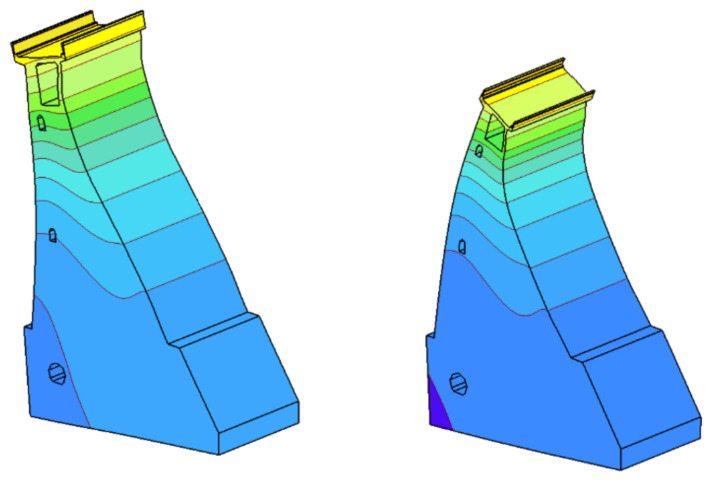
Characteristics of concrete dam displacements during the summer and winter [28]. Blue—the smallest, yellow—the largest displacements.

Exemplary results of the displacements caused by seasonal temperature changes and water fluctuations in the reservoir are shown in Figure 11 in the form of a colored map and a deformed finite element mesh (blue—the smallest displacements and yellow—the largest displacements).

Figure 11 shows that, as a result of high temperatures in the summer, the dam tilts towards the upstream water, and in the winter, when the downstream face is strongly cooled, the dam tilts towards the downstream water. The dam crown experiences the greatest displacements; in the vicinity of the lower gallery, the displacements are zero.

**Figure 12:**
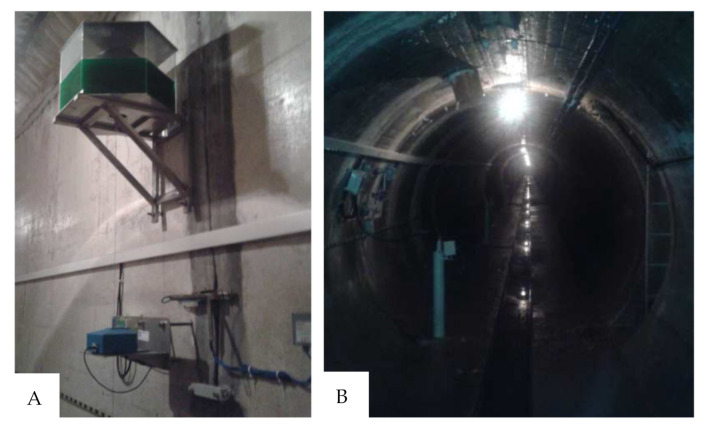
Pendulum for measuring the inclinations (displacement readings point—**A** and pendulum mounting place—**B**).

**Figure 13:**
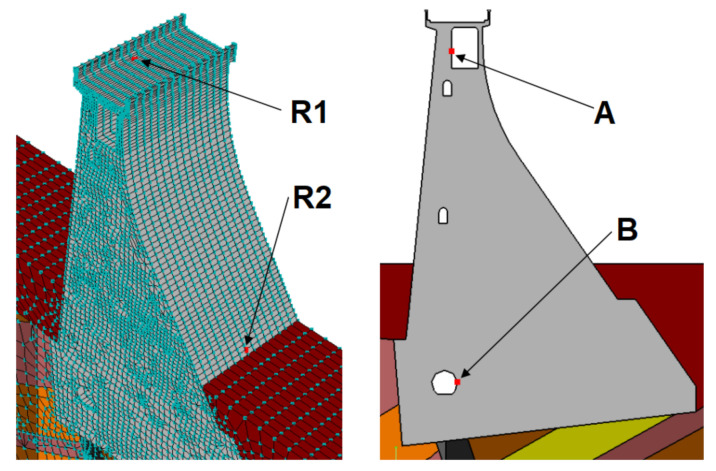
Location of the controlled benchmarks and pendulums in the numerical model. (displacement readings point—A and pendulum mounting place—B, R1 and R2 benchmarks).

**Figure 14:**
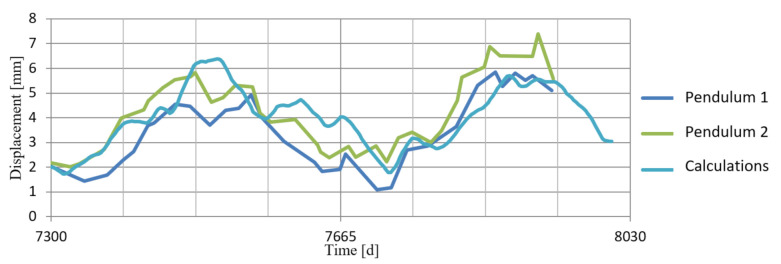
Comparison of the calculation results with the pendulum-measured displacements.

In order to verify the numerical model—i.e., correctness of the chosen geometry, material parameters and forces acting on the dam—the results of the numerical calculations were compared with the displacements of the controlled benchmarks and pendulums installed in the concrete dam section. Figure 12 shows the location of the pendulum (in the lower gallery) and the measuring point (in the upper gallery). Figure 13 shows the locations of these points in the model: point B—place of attachment and point A—place of measurements. The calculation results and the displacement of the pendulum are shown in Figure 14.

Figure 13 also shows the benchmarks (R1 and R2), the displacements of which were compared with the results of the corresponding points in the model. The differential comparison of the benchmark displacements is summarized in Table 1. A high-level correlation was obtained between the results of the calculations and the indications of the control and measurement equipment. This demonstrates the correctness of the assumptions made for the calculations.

Based on a calibrated numerical model, it is possible to analyze the displacements and stresses during the operation of hydrotechnical facilities. The use of numerical models is aimed at improving the safety of facilities and determining the measuring range and threshold values (warning and alarm levels) allowing for early warnings about potentially dangerous situations. Knowing the distribution of the displacements that arise in the structure, it is possible to determine the positioning of additional control and measurement devices.

If it is necessary to extend the monitoring by measuring the tilts of the object in other sections; where the installation of classic pendulums is not possible due to the lack of vertical connection (shafts) between individual galleries, it is possible to install the UMP3 levelers discussed in this article. Based on the analysis of the calculation results, it can be concluded that the best location for placing the precise levelers in the analyzed object so as to verify the calculated concrete dam displacements presented in Figure 11 is the dam’s crown or the highest-located control and measurement gallery.

## 10. Conclusions

The development of measurement methods, methodologies and analyses thereof (including the development of automated systems for the structure condition controls), enables using more effective measurement solutions.

The knowledge of the sensor parameters—in particular, long-term stability—allows for correct inferences with regards to a given structure’s movements and safety.

The solutions presented and results obtained confirm the high level of long-term stability of the sensors, as well as their measurement accuracy.

A high accuracy permits hazardous trends to be detected early enough. Rapidly progressing changes, even if smaller than the accuracy of other sensors, may be a sign of an emergency.

In the case of the system modifications, in order not to lose continuity and correlation with the measurements, one has to apply sensors featuring a similar stability and accuracy.

The results obtained through employing ultrasonic sensors helps to discover subtle structure deformations. This outcome is invaluable when developing and calibrating object models (e.g., by means of the finite element method) and when applying observation methods in structure condition monitoring.

This type of innovative approach not only significantly increases the safety of objects monitored with ultrasonic sensors but also gives a very tangible economic effect, thanks to the avoidance of significant costs associated with the repair of serious damage that often results from the accumulation of minor damage.

A numerical analysis turns out to be a useful asset for studying the behavior of an object that is brought about by various interactions and allows the indication of appropriate locations and the selection of threshold values for the installed control and measurement devices.

## Figures and Tables

**Table 1:** Comparison of the benchmarks (R1 and R2) displacements with the calculation results.

Series	Date	R1 (mm)	R2 (mm)	Calculations (mm)
1	24.02.2010	2.0	1.4	5.2
	07.09.2010	5.8	4.9	8.8
	Difference	3.8	3.5	3.6
2	07.09.2010	5.8	4.9	8.8
	11.03.2011	2.1	1.1	5.1
	Difference	3.7	3.8	3.7
3	11.03.2011	2.1	1.1	5.1
	09.07.2011	7.3	5.8	10.1
	Difference	5.2	4.7	5.0

## Data Availability

www.ultrasystem.eu (accessed on 1 March 2021)
